# Real‐world use of nonvitamin K antagonist oral anticoagulant in atrial fibrillation patients with liver disease: A meta‐analysis

**DOI:** 10.1002/clc.23408

**Published:** 2020-06-17

**Authors:** Qixin Dai, Xiaohong Deng, Lin Zhou, Long Zhang, Xiulin Xiao, Yonghui Liao

**Affiliations:** ^1^ Department of Hepatopancreatobiliary Surgery Ganzhou People's Hospital Ganzhou Jiangxi China

**Keywords:** anticoagulants, atrial fibrillation, liver disease, liver injury, outcome

## Abstract

Several studies have investigated the effectiveness and safety of nonvitamin K antagonist oral anticoagulants (NOACs) vs vitamin K antagonists (VKAs) in patients with atrial fibrillation (AF) and liver disease. Herein, we conducted a meta‐analysis to compare the effect of NOACs with VKAs in patients with AF and liver disease. We also conducted a subsidiary analysis to compare the risk of liver injury between NOACs and VKA in AF patients. We systematically searched the PubMed and Embase databases from January 2009 to May 2020 for the relevant studies. Hazard ratios (HRs) with 95% confidence intervals (CIs) were selected and pooled using a random‐effects model. A total of six cohorts were included. Compared with VKA use, the use of NOACs was associated with reduced risks of stroke or systemic embolism (HR 0.68, 95% CI 0.49‐0.93), all‐cause death (HR 0.69, 95% CI 0.63‐0.75), and intracranial bleeding (HR 0.49, 95% CI 0.40‐0.59), whereas the outcomes of major bleeding (HR 0.72, 95% CI 0.51‐1.01) and gastrointestinal bleeding (HR 0.84, 95% CI 0.51‐1.36) were not significantly different between groups in AF patients with liver disease. Moreover, compared with VKA use, the use of NOACs was associated with a reduced risk of liver injury (HR 0.72, 95% CI 0.61‐0.84) in AF patients. Compared with VKAs, the use of NOACs was associated with reduced risks of stroke or systemic embolism, all‐cause death, and intracranial bleeding in AF patients with liver disease, and associated with a reduced risk of liver injury in AF patients.

## 1 INTRODUCTION

Atrial fibrillation (AF) represents one of the most common arrhythmias, resulting in an increased risk of thromboembolic events.^1^ Current guidelines recommend appropriate thromboprophylaxis with oral anticoagulants for stroke prevention in patients with AF.^2^, ^3^ Nonvitamin K antagonist oral anticoagulants (NOACs) could be the first choice in nonvalvular AF patients based on evidence from phase III randomized clinical trials.^4^, ^5^, ^6^, ^7^, ^8^ However, in some of these NOAC trials, patients with liver disease were excluded during the assessment of NOACs in AF. Therefore, the effectiveness and safety of NOACs compared with vitamin K antagonists (VKAs) are less clear among AF patients with liver disease. To date, several studies regarding this issue have been published,^9^, ^10^, ^11^, ^12^, ^13^, ^14^ but their findings are inconsistent. A previous meta‐analysis by including these studies has indicated that the use of NOACs compared with warfarin is associated with decreased risks of all‐cause death, major bleeding and intracranial bleeding, but they had a similar risk of stroke or systemic embolism and gastrointestinal bleeding in AF patients with liver disease.^15^ This study also included the data of randomized clinical trial^12^ or the unadjusted data.^13^ Therefore, the first section of our meta‐analysis aimed to assess the use of NOACs vs VKAs in AF patients with liver disease by only including the real‐world studies.

Emerging pieces of evidence from case reports and pharmacovigilance analyses have detected a hepatotoxic potential in the NOAC users.^16^, ^17^ Current guidelines recommend annual monitoring of liver function during the use of NOACs.^2^, ^3^ More recently, two observational studies^18^, ^19^ have assessed the risk of liver injury associated with the use of NOACs. Herein, the second section of this meta‐analysis aimed to explore the risk of liver injury of NOACs compared with VKAs in AF patients.

## 2 METHODS

The findings of this meta‐analysis were reported based on the Preferred Reporting Items for Reporting Systematic Reviews and Meta‐analyses (PRISMA).^20^

### 2.1 Aims and eligibility criteria

The objectives of this meta‐analysis were (a) to compare the effectiveness and safety outcomes between NOACs vs warfarin in AF patients with liver disease and (b) to examine the risk of liver injury of NOACs compared with VKAs in AF patients with or without liver disease. We included the studies if they satisfied the following criteria: (a) design of the study: observational studies; (b) comparisons of the study: any NOAC (dabigatran, rivaroxaban, edoxaban or apixaban) vs warfarin; and (c) the effectiveness outcomes including stroke or systemic embolism, and all‐cause death; and the safety outcomes including major bleeding, gastrointestinal bleeding, and intracranial bleeding. We excluded certain types of publications such as abstracts, reviews, editorials, letters to editors, comments, and nonhuman studies.

### 2.2 Literature search

We systematically searched the PubMed and Embase databases from January 2009 to May 2020 because the first publication of NOAC (dabigatran) in AF patients was reported in 2009. The search strategies in the PubMed database is shown in Table S1. The search with keywords were performed by including the following terms: (a) atrial fibrillation; AND (b) nonvitamin K antagonists OR new oral anticoagulants OR novel oral anticoagulants OR direct oral anticoagulants OR oral thrombin inhibitors OR oral factor Xa inhibitors OR dabigatran OR rivaroxaban OR apixaban OR edoxaban; (c) vitamin K antagonists OR warfarin OR coumadin OR acenocoumarol OR phenprocoumon; AND liver disease OR impaired liver disease OR cirrhosis OR liver dysfunction OR liver injury. In addition, we searched the reference lists of previous reviews^21^, ^22^, ^23^ to identify additional publications. There were no language restrictions were applied during the searching process.

### 2.3 Study selection and data abstraction

Two authors (Qixin Dai and Xiaohong Deng) independently screened all of the studies retrieved by the search strategy. According to the inclusion and exclusion criteria, the first phase was to find out the potentially available studies by screening the titles and/or abstracts. The second phase was to read the full text in more details and decide which study could be included. If facing the disagreements in the process, they would solve with it by a discussion with each other, or ask for help from the third author (Yonghui Liao).

We included the following information in each included study: the first author and publication year, study design, data source, inclusion criteria, age and sex, the total number of patients, follow‐up time, definitions of liver disease, effectiveness and safety outcomes. The adjusted hazard ratios (HRs) and 95% confidence intervals (CIs) were regarded as the effect estimates. If the HRs were reported using multiple adjusted models, the most adjusted one was abstracted.

### 2.4 Quality assessment

The quality of the included studies was evaluated by the Newcastle‐Ottawa Scale (NOS)^24^ by two authors (Qixin Dai and Xiaohong Deng) independently. This scale mainly included the three parts, namely selection of cohorts, comparability of cohorts, and assessments of the outcome. A NOS of ≥6 points indicated a moderate‐to‐high quality, whereas a NOS of <6 points indicated a low quality.^15^, ^25^

### 2.5 Statistical analysis

Statistical analysis was performed using the Review Manager Version 5.3 (the Nordic Cochrane Center, Rigshospitalet, Denmark; https://ims.cochrane.org/revman). For each study, we calculated the natural logarithm of the HR (Ln[HR]) and its corresponding SE (SE_Ln[HR]_).^26^ Ln[HR] and SE_Ln[HR]_ were pooled by a random‐effects model weighted by the inverse‐variance method. The Cochrane Q test and *I* ^2^ statistic were used to evaluate heterogeneity, where *P* <.1 and *I* ^2^ >50% indicated a substantial heterogeneity, respectively. In the sensitivity analysis, we separately reported the effectiveness and safety of NOACs and VKAs in AF patients with cirrhosis. We also performed the subgroup analysis based on the type of NOACs. It was unsuitable to examine the publication bias when the number of included studies was less than 10. The statistical significance threshold was set at *P* <.05.

## 3 RESULTS

### 3.1 Study selection

**FIGURE 1:**
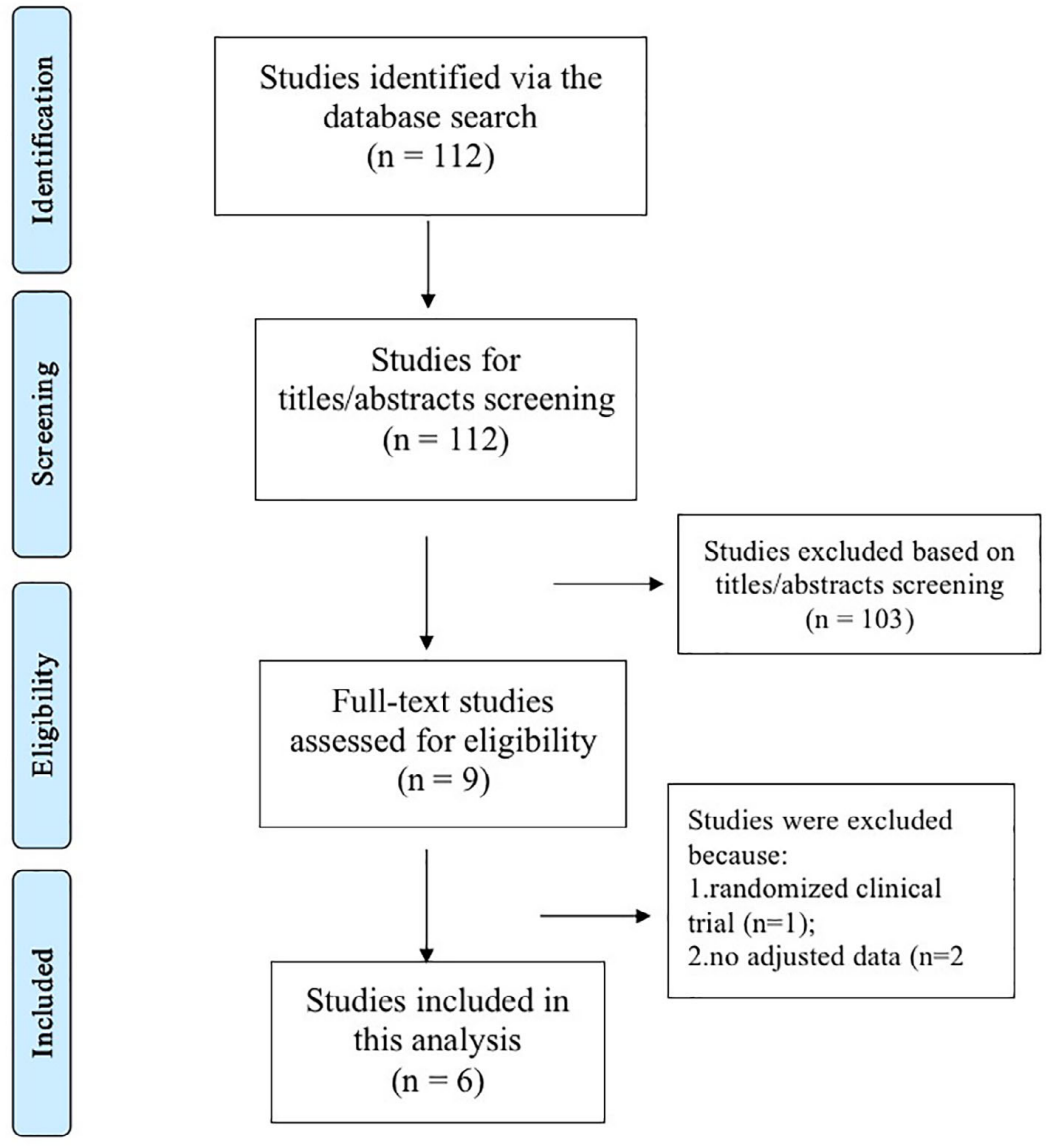
Overview of the research strategy

The literature retrieval process is presented in Figure 1. We initially identified 112 studies through the electronic searches in the PubMed and Embase databases. We found no additional studies through the reference lists of previous reviews.^21^, ^22^, ^23^ Based on the title‐/abstract‐ screenings, 103 studies were excluded because they had no relevant data. The nine remaining studies were reviewed in more detail and three studies were excluded because: (a) two studies did not report adjusted HRs,^13^, ^27^ and (b) one study was not an observational cohort.^12^ Finally, a total of six observational cohorts were included for our quantitative analysis.^9^, ^10^, ^11^, ^14^, ^18^, ^19^ The baseline characteristics of the included studies are shown in Table 1. All the included studies had a NOS score of ≥6 points (Table 1).

**TABLE 1:** Baseline characteristics of the included studies

Study (first author‐year)	Study type	Populations	Data source	Age/sex	NOACs presented	Follow‐up	Study quality
Douros‐2018	Observational cohort	Patients newly diagnosed with AF taking apixaban, dabigatran, rivaroxaban, or VKAs	Administrative databases of the Canadian province of Quebec's health insurances	76.1/both	Apixaban, dabigatran, rivaroxaban; unknown dose	NA	8 stars
Alonso‐2017	Observational cohort	Patients with AF taking apixaban, dabigatran, rivaroxaban, or warfarin	MarketScan Commercial and Medicare Supplemental databases from November 4, 2011 to December 31, 2014	70.0/both	Apixaban, dabigatran, rivaroxaban; unknown dose	1.0 y	8 stars
Lee‐2019	Observational cohort	Liver cirrhotic patients with AF taking apixaban, dabigatran, rivaroxaban, or warfarin	Taiwan National Health Insurance Research Database from June 1, 2012, to December 31, 2016	72.6/both	Apixaban, dabigatran, rivaroxaban; both low and standard dose	NOACs:1.13 y Warfarin:1.30 y	8 stars
Lee‐2019	Observational cohort	Advanced liver disease patients with AF taking apixaban, dabigatran, rivaroxaban, edoxaban, or warfarin	Korean National Health Insurance Service database	69.0/both	Apixaban, dabigatran, rivaroxaban; edoxaban; both low and standard dose	Mean 1.2 y	8 stars
Goriacko‐2018	Observational cohort	AF patients with chronic liver disease taking apixaban, dabigatran, rivaroxaban, edoxaban, or warfarin	An urban academic health system from May 1, 2009 to May 1, 2016	65.3/both	NA	NA	7 stars
Wang‐2018	Observational cohort	AF patients with impaired liver function taking apixaban, dabigatran, rivaroxaban, edoxaban, or warfarin	Electronic medical records conducted from 2009 to 2016 at a multicenter healthcare provider in Taiwan	77.3/both	Apixaban, dabigatran, rivaroxaban; edoxaban; unknown dose	NA	7 stars

Abbreviations: AF, atrial fibrillation; NA, not available; NOACs, nonvitamin K antagonist oral anticoagulants; VKAs, vitamin K antagonists.

### 3.2 Effectiveness and safety of NOACs vs VKAs in AF patients with liver disease

**FIGURE 2:**
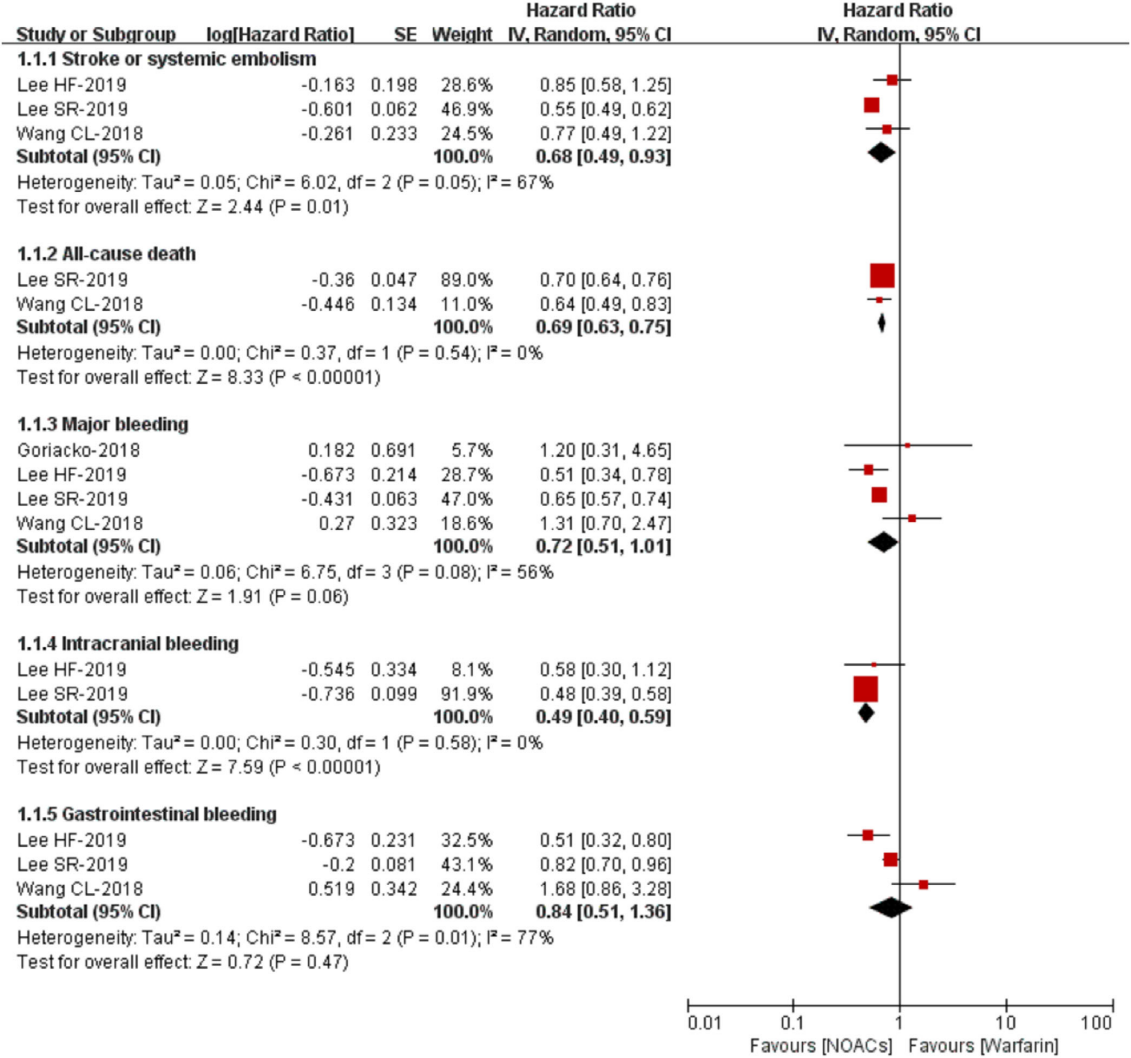
Hazard ratios of effectiveness and safety outcomes for NOACs compared with VKAs in AF patients with liver diseases. AF, atrial fibrillation; CI, confidence interval; IV, inverse of the variance; NOACs, nonvitamin K antagonist oral anticoagulants; SE, standard error; VKAs, vitamin K antagonists

Four studies assessed the effectiveness and safety of NOACs vs VKAs in AF patients with liver disease. For the effectiveness outcomes, as shown in Figure 2, compared with VKA use, the use of NOACs was associated with reduced risks of stroke or systemic embolism (HR 0.68, 95% CI 0.49‐0.93) and all‐cause death (HR 0.69, 95% CI 0.63‐0.75). For the safety outcomes, as presented in Figure 2, compared with VKA use, the use of NOACs was associated with a decreased risk of intracranial bleeding (HR 0.49, 95% CI 0.40‐0.59). However, the safety outcomes of major bleeding (HR 0.72, 95% CI 0.51‐1.01) and gastrointestinal bleeding (HR 0.84, 95% CI 0.51‐1.36) were not significantly different between NOACs vs VKAs.

#### 3.2.1 Sensitivity analysis and subgroup analysis

Two included studies reported the effectiveness and safety of NOACs and VKAs in AF patients with cirrhosis. As presented in Table 1, compared with VKA use, the use of NOACs was associated with reduced risks of all‐cause death (HR 0.70, 95% CI 0.64‐0.76), major bleeding (HR 0.53, 95% CI 0.37‐0.76), intracranial bleeding (HR 0.55, 95% CI 0.31‐0.97), and gastrointestinal bleeding (HR 0.57, 95% CI 0.38‐0.84). There was no difference in the risk of stroke or systemic embolism (HR 0.81, 95% CI 0.57‐1.15) between NOACs vs VKAs. In addition, we performed the subgroup analysis based on the NOACs type, suggesting that all NOACs (dabigatran, rivaroxaban, edoxaban, or apixaban) had lower or similar risks of thromboembolic and bleeding events compared with VKAs in AF patients with liver disease (Table 1).

### 3.3 Risk of liver injury between NOACs vs VKAs in AF patients

Two included studies assessed the risk of liver injury between NOACs vs VKAs in AF patients with or without liver disease.^18^, ^19^ As shown in Figure S1, compared with VKA use, the use of NOACs was associated with a reduced risk of liver injury (HR 0.67, 95% CI 0.56‐0.80).

#### 3.3.1 Subgroup analysis

We performed the subgroup analysis based on the NOACs type, suggesting that compared with VKA use, the use of dabigatran (HR 0.54, 95% CI 0.44‐0.67), rivaroxaban (HR 0.82, 95% CI 0.70‐0.96), or apixaban (HR 0.65, 95% CI 0.45‐0.95) had a lower risk of liver injury in AF patients (Figure S2).

## 4 DISCUSSION

### 4.1 Main findings

In the present meta‐analysis, our data indicated that compared with VKA use (a) the use of NOACs was associated with reduced risks of stroke or systemic embolism, all‐cause death, and intracranial bleeding. There was no significant difference in major or gastrointestinal bleeding between the two studied groups; and (b) the use of NOACs was associated with a reduced risk of liver injury in AF patients.

### 4.2 Comparison with other studies

Advanced liver diseases, such as acute or chronic hepatitis and cirrhosis, or elevation of liver enzymes, are known to increase the risks of stroke or systemic embolism and bleeding events. As such, these patients should receive therapy with oral anticoagulants including NOACs or VKAs. One previous systematic review has evaluated the effectiveness and safety of NOACs in cirrhosis patients with venous thromboembolism, splanchnic vein thrombosis, or AF.^22^ However, cirrhosis patients with AF were not analyzed separately in this descriptive analysis.^22^ Although two prior meta‐analyses assessed the effect of NOACs compared with VKAs in AF patients with liver disease, they still had several defects, such as including the unadjusted data,^15^, ^23^ or combining data of real‐world settings and randomized clinical trials,^15^ which might influence the validity of findings. In addition, Chokesuwattanaskul et al^23^ only included two studies for analysis. Caldeira et al^21^ only included the randomized clinical trials, and focused on all patients with NOACs. In contrast, our current meta‐analysis only included adjusted data of real‐world studies to compare the effectiveness and safety of NOACs and VKAs in AF patients with liver disease, suggesting that NOACs had lower or similar rates of thromboembolic and bleeding events compared with VKAs. The subgroup analysis based on the NOAC type indicated similar results with the primary analysis.

Several studies have examined the hepatotoxic potential of NOACs, but their findings are inconsistent.^16^, ^18^, ^19^, ^28^ Case reports and analyses of pharmacovigilance data have found an increased risk of liver injury during the use of NOACs, especially for rivaroxaban.^16^, ^28^ For the data in the pharmacovigilance databases, there have many limitations such as under‐reporting of adverse outcomes, selective increased reporting for NOACs, and incomplete data.^29^ Population‐based studies could provide more detail regarding the hepatic safety of NOACs. To date, two observational studies have assessed the risk of liver injury associated with the use of NOACs compared with VKAs.^18^, ^19^ After pooling these two studies, we first found that the use of NOACs (regardless of the NOAC type) vs VKAs was associated with a reduced risk of liver injury in AF patients (Table 2).

**TABLE 2:** HRs of effectiveness and safety outcomes between NOACs vs VKAs in AF patients with liver diseases

	Stroke or systemic embolism	All‐cause death	Major bleeding	Intracranial bleeding	Gastrointestinal bleeding
Patients with liver diseases	0.68 (95%CI: 0.49–0.93)	0.69 (95%CI: 0.63–0.75)	0.72 (95%CI: 0.51‐1.01)	0.49 (95%CI: 0.40‐0.59)	0.84 (95%CI: 0.51‐1.36)
Patients with cirrhosis	0.81 (95%CI: 0.57‐1.15)	0.70 (95%CI: 0.64‐0.76)	0.53 (95%CI: 0.37‐0.76)	0.55 (95%CI: 0.31‐0.97)	0.57 (95%CI: 0.38‐0.84)
NOAC type					
*Dabigatran*	0.68 (95%CI: 0.42‐1.11)	0.64 (95%CI: 0.55‐0.73)	0.53 (95%CI: 0.44‐0.63)	0.38 (95%CI: 0.28‐0.52)	0.66 (95%CI: 0.53‐0.83)
*Rivaroxaban*	0.71 (95%CI: 0.42‐1.21)	0.79 (95%CI: 0.70‐0.89)	0.57 (95%CI: 0.28‐1.15)	0.54 (95%CI: 0.42‐0.69)	0.64 (95%CI: 0.26‐1.61)
*Apixaban*	0.60 (95%CI: 0.24‐1.53)	0.85 (95%CI: 0.73‐0.99)	0.60 (95%CI: 0.49‐0.74)	0.54 (95%CI: 0.39‐0.75)	0.67 (95%CI: 0.51‐0.88)
*Edoxaban*	0.86 (95%CI: 0.46‐1.61)	0.49 (95%CI: 0.28‐0.78)	0.62 (95%CI: 0.40‐0.96)	0.88 (95%CI: 0.39‐1.99)	0.48 (95%CI: 0.23‐1.00)

Abbreviations: AF, atrial fibrillation; CI, confidence interval; HR, hazard ratio; NOACs, nonvitamin K antagonist oral anticoagulants; VKAs, vitamin K antagonists.

### 4.3 Implications and further research

Until head‐to‐head prospective randomized trials that reflect routine use of NOACs in AF patients with liver disease are available, our comparisons based on real‐world studies might help clinicians in decision‐making for the choice of anticoagulants for stroke prevention in this population. Nevertheless, the residual confounders from unmeasured factors might influence the validity of our findings due the nature of observational data. There is still an increased need for more studies to confirm our findings.

### 4.4 Strengths and limitations of study

An obvious strength of this study was inclusion of only studies that reported adjusted results in the pooled analysis. In addition, this was the first study to assess the risk of liver injury of NOACs with VKAs in patients with AF. Nevertheless, several limitations should be acknowledged. First, this study was performed based on the observational data, the residual confounders from unmeasured factors might influence the validity of our findings. In addition, The protocol of the systematic review and meta‐analysis was not registered in the PROSPERO database. Second, the definitions of liver disease were different across the included studies, which could affect the subsequent outcomes. Third, the data about patient adherence and persistence of anticoagulants was unavailable. Fourth, the publication bias could be done because of the limited number of studies. Finally, the number of included studies in some comparisons was small, limiting the validity of the corresponding findings.

## 5 CONCLUSIONS

Compared with VKAs, the use of NOACs was associated with reduced risks of stroke or systemic embolism, all‐cause death, and intracranial bleeding in AF patients with concomitant liver disease, and associated with a reduced risk of liver injury in AF patients.

## CONFLICT OF INTEREST

The authors declare that they have no conflict of interest.

## AUTHOR CONTRIBUTIONS

Qixin Dai and Xiaohong Deng performed the literature search, data extraction, statistical analysis, and writing the original manuscript. Xiulin Xiao and Yonghui Liao help revise the article. Lin Zhou and Long Zhang checked the data.

## Supporting information


**Appendix** **S1**: Supporting Information
