# Supplementary material for: Real‐world use of nonvitamin K antagonist oral anticoagulant in atrial fibrillation patients with liver disease: A meta‐analysis
Source: Clin Cardiol. 2020 Jun 17;43(7):676–83. doi: 10.1002/clc.23408 (PMC7368301; doi:10.1002/clc.23408)
Supplement: Supplementary file 1 — Appendix S1: Supporting Information [file CLC-43-676-s001.docx]

**Supplementary Table 1. The search strategies of this meta-analysis**

|  | **Searching terms** | **No. of studies** |
| --- | --- | --- |
| (1) | atrial fibrillation | 82555 |
| (2) | non-vitamin K antagonists OR new oral anticoagulants OR novel oral anticoagulants OR direct oral anticoagulants OR oral thrombin inhibitors OR oral factor Xa inhibitors OR dabigatran OR rivaroxaban OR apixaban OR edoxaban | 17977 |
| (3) | vitamin K antagonists OR warfarin OR coumadin OR acenocoumarol OR phenprocoumon | 38472 |
| (4) | liver disease OR impaired liver disease OR cirrhosis OR liver dysfunction OR liver injury | 966844 |
| (5) | Combine (1) to (4), from January 2009 to February 2020 | 106 |


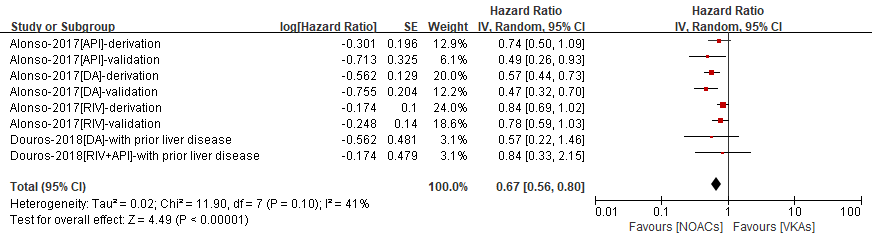


**Supplementary Figure 1. Hazard ratios of liver injury for NOACs compared with VKAs in AF patients**

**AF = atrial fibrillation; NOACs = non-Vitamin K antagonist oral anticoagulants; VKAs= vitamin K antagonists; CI = confidence interval; SE = standard error; IV = inverse of the variance.**


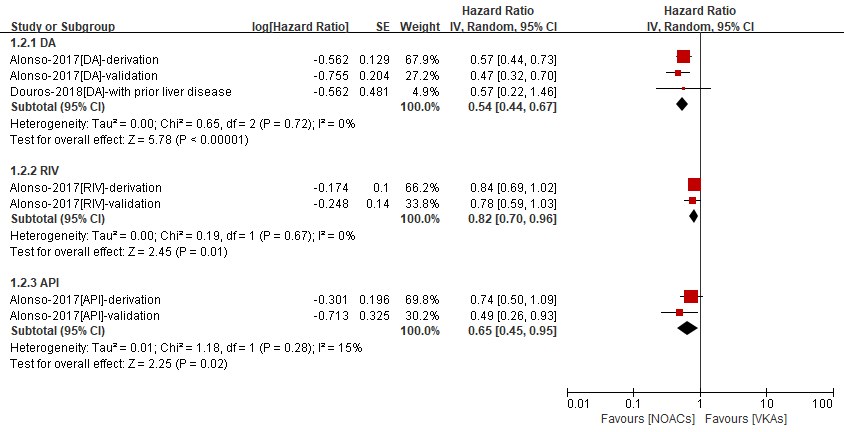


**Supplementary Figure 2. Hazard ratios of liver injury for each NOAC compared with VKAs in AF patients**

**AF = atrial fibrillation; NOACs = non-Vitamin K antagonist oral anticoagulants; VKAs= vitamin K antagonists; CI = confidence interval; SE = standard error; IV = inverse of the variance.**
